# Regional differences and spatial patterns of health status of the member states in the “Belt and Road” Initiative

**DOI:** 10.1371/journal.pone.0211264

**Published:** 2019-01-30

**Authors:** Jie Li, Fangjin Xu, Zhaojun Sun, Jinfeng Wang

**Affiliations:** 1 Department of Resources and Environment, Ningxia University, Yinchuan, Ningxia, China; 2 Ningxia (China-Arab) Key Laboratory of Resource Assessment and Environmental Regulation in Arid Region, Ningxia University, Yinchuan, Ningxia, China; 3 State Key Laboratory of Resources and Environmental Information System, Institute of Geographic Sciences and Natural Resources Research, Chinese Academy of Sciences, Beijing, China; University of Ottawa, CANADA

## Abstract

The strategy of the “Belt and Road” initiative aims not only to promote the cooperation and the development of economic trade, but also to boost the integration and development in multiple fields—especially in the field of health. This paper explores the health levels of member-states in the Belt and Road initiative from the perspective of regional differences and spatial patterns. Data from the 68 member-states in the Belt and Road initiative were selected from the statistical data on disease and socioeconomics in all countries from the 2015 publication by the World Bank and the World Health Organization. Health indicators that can reflect health levels of member states were selected. Moran’s I and Getis-Ord Gi* were used to analyze the spatial clustering and hot/cold spots of the health status. After that a novel spatial statistical method “geographical detector” was used to analysis the spatial stratified heterogeneity of the selected health indicators. The result showed that the health level of the member states fluctuated around the world average and varied greatly within the member states. The health status of the member states showed spatial clustering, and the q-statistics of the geographical detector confirmed that the health status demonstrated statistically significant spatial heterogeneity for different continent the member states reside. In general, member states in Europe and Oceania demonstrated higher health status, while those in South Asia, Southeast Asia, Africa and part of Middle East have lower health status. In particular, special attention should be paid to control communicable diseases in African member states. Different regions and member states face different kinds of health threats in various degrees. Member states should strengthen health cooperation between themselves and work closely with other countries to make the “belt and road” a healthy road.

## Introduction

The “belt and road” initiative refers to the Silk Road Economic Belt and the 21st Century Maritime Silk Road. It is a strategic concept proposed by Chinese President Xi Jinping in September and October 2013. It is highly regarded by the international community, with the relevant countries also responding positively. The “belt and road” construction is a systematic project, its main purpose is not only to carry out pragmatic cooperation in key areas such as interoperability, capacity cooperation, and trade and investment, but also to promote the development of various forms of higher-level operations: thus, achieving common prosperity and mutual benefit [1, 2]. Health is an indispensable requirement for the promotion of humanities’ all-round development and is a basic condition for economic and social development. Health cooperation is an important part of the “belt and road” initiative [3].

The “belt and road” initiative covers 68 countries in Asia Pacific, Europe, the Central East, Africa, and the South Pacific, with an overall population over 4.4 billion. This paper aims to summarize the health status of the 68 countries by analyzing the health indicators of member states and provide a scientific basis for the comprehensive construction of the “belt and road” initiative [4].

In this paper, we selected six health indicators to study. We had three central aims. The first aim was to illustrate the regional differences between the six indicators to make a comparison between countries among the Belt and Road initiative and the world average, to explore the overall health level among the Belt and Road participants. Secondly, the spatial distribution patterns of each health indicator are found by Moran's I and Getis-Ord Gi*. Finally, based on this data, we summarize characteristics and identify health problems of each region within the Belt and Road initiative.

## Area, methods and data sources

### Research area

**Fig 1:**
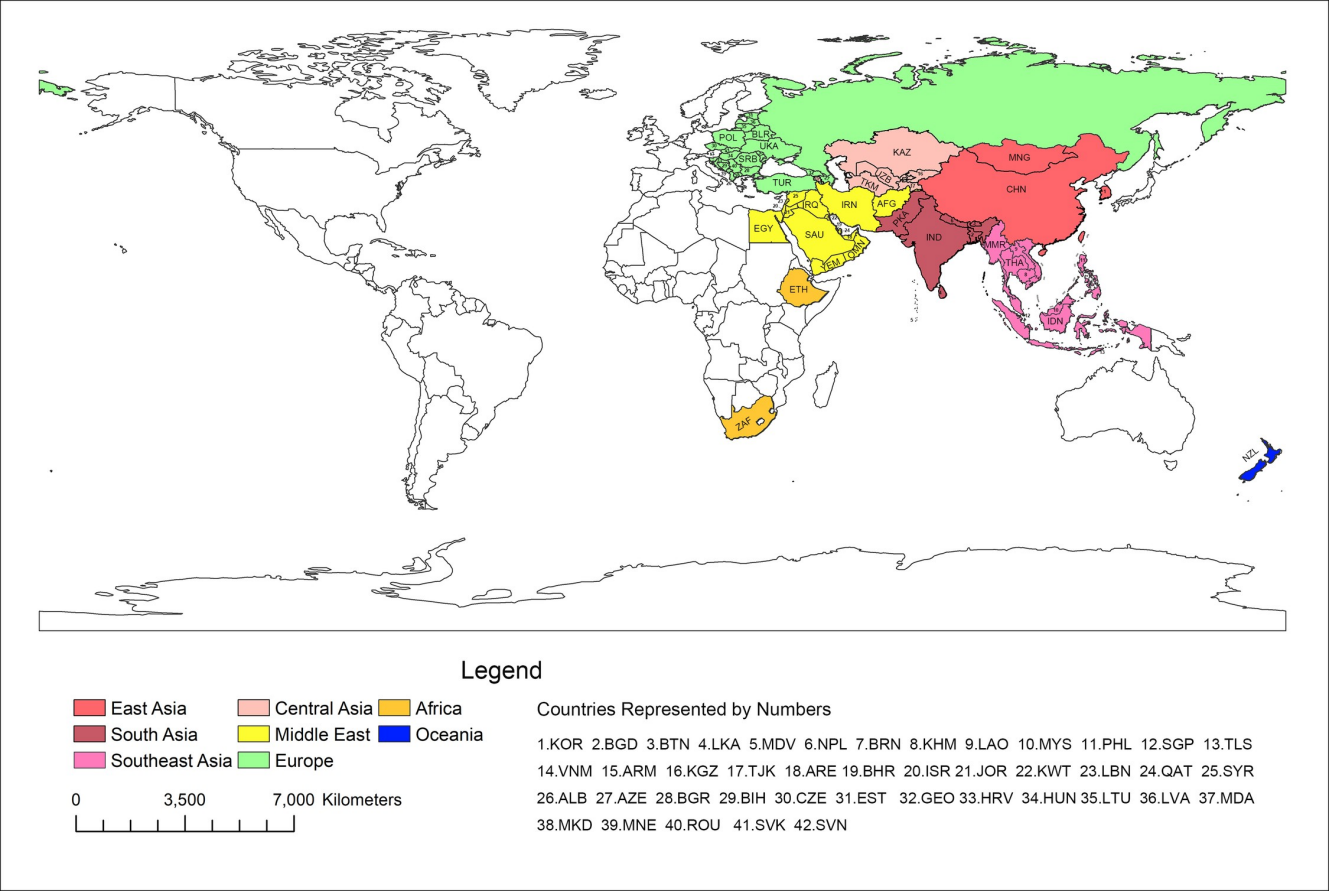
Geographic distribution of the member states of the belt and road” initiative.

By March 2018, 68 countries had joined the “belt and road” initiative (hereinafter referred to as the member states). All of the member states were selected for analysis in this paper. They include East Asia, South Asia, Southeast Asia, Central Asia, Middle East, Africa, Europe and Oceania, according to the zoning of the World Bank [5] and geopolitical factors [6, 7]. The majority of the member states are located in Asia, Europe and the Middle East (Table 1). The geographical distribution of member states is shown in Fig 1.

**Table 1:** Member states of the “belt and road” initiative (up to March, 2018).

Region		Member states
Asia	East Asia	China (CHN), Korea (KOR), Mongolia (MNG),
	South Asia	Bangladesh (BGD), Bhutan (BTN), India (IDN), Sri Lanka (LAK), Maldives (MDV), Nepal (NPL), Pakistan (PAK)
	Southeast Asia	Brunei Darussalam (BRN), Indonesia (IDN), Cambodia (KHM), Lao PDR (LAO), Myanmar (MMR), Malaysia (MYS), Philippines (PHL), Singapore (SGP), Thailand (THA), Timor-Leste (TLS), Vietnam (VNM)
	Central Asia	Armenia (ARM), Kazakhstan (KAZ), Kyrgyz Republic (KGZ), Tajikistan (TJK), Turkmenistan (TKM), Uzbekistan (UZB)
Middle East		Afghanistan (AFG), United Arab Emirates (ARE), Bahrain (BHR), Egypt, Arab Rep. (EGY), Iran Islamic Rep. (IRN), Iraq (IRQ), Israel (ISR), Jordan (JOR), Kuwait (KWT), Lebanon (LBN), Oman (OMN), Qatar (QAT), Saudi Arabia (SYR), Syrian Arab Republic (SAU), Yemen, Rep. (YEM)
Europe		Albania (ALB), Azerbaijan (AZE), Bulgaria (BGR), Bosnia and Herzegovina (BIH), Belarus (BLR), Czech Republic (CZE), Estonia (EST), Georgia (GEO), Croatia (HRV), Hungary (HUN), Lithuania (LTU), Latvia (LVA), Moldova (MDA), Macedonia, FYR (MKD), Montenegro (MNE), Poland (POL), Romania (ROU), Russian Federation (RUS), Serbia (SRB), Slovak Republic (SVK), Slovenia (SVN), Turkey (TUR), Ukraine (UKR)
Africa		Ethiopia (ETH), South Africa (ZAF)
Oceania		New Zealand (NZL)

### Selection of health indicators

Health status is a collective result of complex interactions between many physical geographic (e.g., temperature, precipitation, altitude) and socioeconomic (e.g., accessibility, quality of healthcare, and cultural and normative factors associated with healthy lifestyles and dietary patterns). In order to measure the health status of the member states and compare them to other countries in a global scale in an objective and universally applicable manner, health indicators from the 2018 Global Reference List of 100 Core Health Indicators (referred to hereafter as “The Global Reference List”) published by World Health Organization (WHO) were utilized[8]. An indicator is prioritized as “core” if it meets all of the following four criteria[8]:

1. The agreement of all member states of WHO,
2. Scientifically robust, useful, accessible, understandable as well as specific, measurable, achievable, relevant and time-bound (SMART),
3. Extensive measurement experience supported by an international database,
4. The indicator is being used by countries in the monitoring of national plans and programmes.

As such, the Global Reference List is a standard set of core indicators prioritized by the global community to provide concise information on the health situation at national and global levels[8]. The list includes a selection of priority indicators relating to four domains that includes health status, risk factors, service coverage and health systems[8]. As the main purpose of this paper is to present the current health status of the member states, only indicators in the health status category are selected. There are four sections in the health status category, within each section multiple indicators are present detailing different aspects of health status (Table 2). Although different, the health status information manifested by the indicators in each section contains some overlaps in a global scale. Therefore, six indicators that are representative of each section are selected (bold font text in Table 2), including[8]:

- Life expectancy at birth

Life expectancy at birth describes the possible lifespan of an infant considering the influence of age and gender-related lethal factors[8]. It is a statistic based on current age, birth year, and other factors that influence demographic change Life expectancy can be used to compare health between countries, or to measure changes over time. These comparisons can inform policy questions that depend on how morbidity changes as mortality decreases [9]. Life expectance has been used extensively to evaluate the health status of countries worldwide [9–11].

- Maternal mortality

Maternal death includes death of women during pregnancy, production, and within 42 days of birth [12]. Maternal health level varies greatly by area [13]. In 2015, the risk of maternal death in developing countries was about 33 times higher than that in developed countries [14]. Considering that both developed and developing countries are present as the member states, maternal mortality was selected to shed more light on the spatial variability of the health status within the member states.

- Premature non-communicable disease (NCD) mortality

Premature NCD mortality is the mortality rate caused by cardiovascular diseases, cancer, diabetes, or chronic respiratory diseases from ages 30 to 70 years. Due to the changes in people's life and work patterns, chronic diseases such as cardiovascular disease, cancer, and death and disabilities caused by chronic noncommunicable disease have become one of the threats to human health worldwide [15]. This indicator was chosen as vital complementary information for the morbidity caused by infectious diseases such as TB and HIV, which were also selected as health indicators in this paper. Together, they give comprehensive information on health status of a country from the perspective of the prevalence of diseases, whether non-communicable or infectious.

- Total fertility rate

Total fertility rate can be expressed by the births per woman [16]. It is a comprehensive factor reflecting cultural and socioeconomic characteristics of a country, such as maternal age during the first childbirth, residential area of the women, religion, monthly household income, perceptions about the value of marriage and children, and social media[17]. Total fertility rate can also reflect nutrition, quality and safety of heal care, and utilization and access of health care services of a country. It was selected to provide additional information on health status together with other selected health indicators.

- Human Immunodeficiency Virus (HIV) prevalence rate

HIV prevalence rate was selected to reflect the prevalence of sexually transmitted infectious diseases in a country. HIV has begun to spread rapidly to developing countries in the context of globalization. The amount of an HIV-infected population varies geographically, areas in South Asia, Southeast Asia, and Sub-Saharan Africa have been reported with high HIV infection rates [18].

- Tuberculosis (TB) incidence rate

TB incidence rate is in a given year, the estimated number of new and relapse-TB cases expressed as a rate per 100 000 population [16]. TB is the ninth leading cause of death worldwide from a single infectious disease [19]. TB incidence rate was selected, together with HIV, to represent prevalence rate of infectious diseases in a country.

**Table 2:** Indicators in the health status category. Indicators selected to reflect the health status are in bold font.

Health status	Indicators
Mortality by age and sex	**Life expectancy at birth**, Adolescent mortality rate; adult mortality rate between 15 and 60 years of age; under-five mortality rate; infant mortality rate; neonatal mortality rate; stillbirth rate.
Mortality by cause	**Maternal mortality ratio**, TB mortality rate; AIDS-related mortality rate; Malaria mortality rate; **Premature noncommunicable disease (NCD) mortality**; mortality from household and ambient air pollution; mortality from unsafe water; unsafe sanitation and lack of hygiene; mortality from unintentional poisoning; suicide rate; death rate due to road traffic injuries; number of deaths; missing persons and persons affected by disaster per 100 000 people; mortality rate due to homicide.
Fertility	Adolescent birth rate, **Total fertility rate**
Morbidity	New cases of vaccine-preventable diseases; new cases of IHR-notifiable diseases and other notifiable diseases; **HIV prevalence rate;** HIV incidence rate; Hepatitis B surface antigen prevalence; Hepatitis B incidence; sexually transmitted infections (STIs) incidence rate; congenital syphilis rate; **TB incidence rate;** TB notification rate; Malaria parasite prevalence among children aged 6–59 months; Malaria incidence rate; cancer incidence, by type of cancer.

### Data analysis

The data analysis can be divided into two parts. The first part employed exploratory spatial data analysis techniques to reveal the distribution of the health status of member countries. In the second part a novel statistical method “geographical detector” was used to test the stratified spatial heterogeneity found by visual inspection during exploratory spatial data analysis.

**Fig 2:**
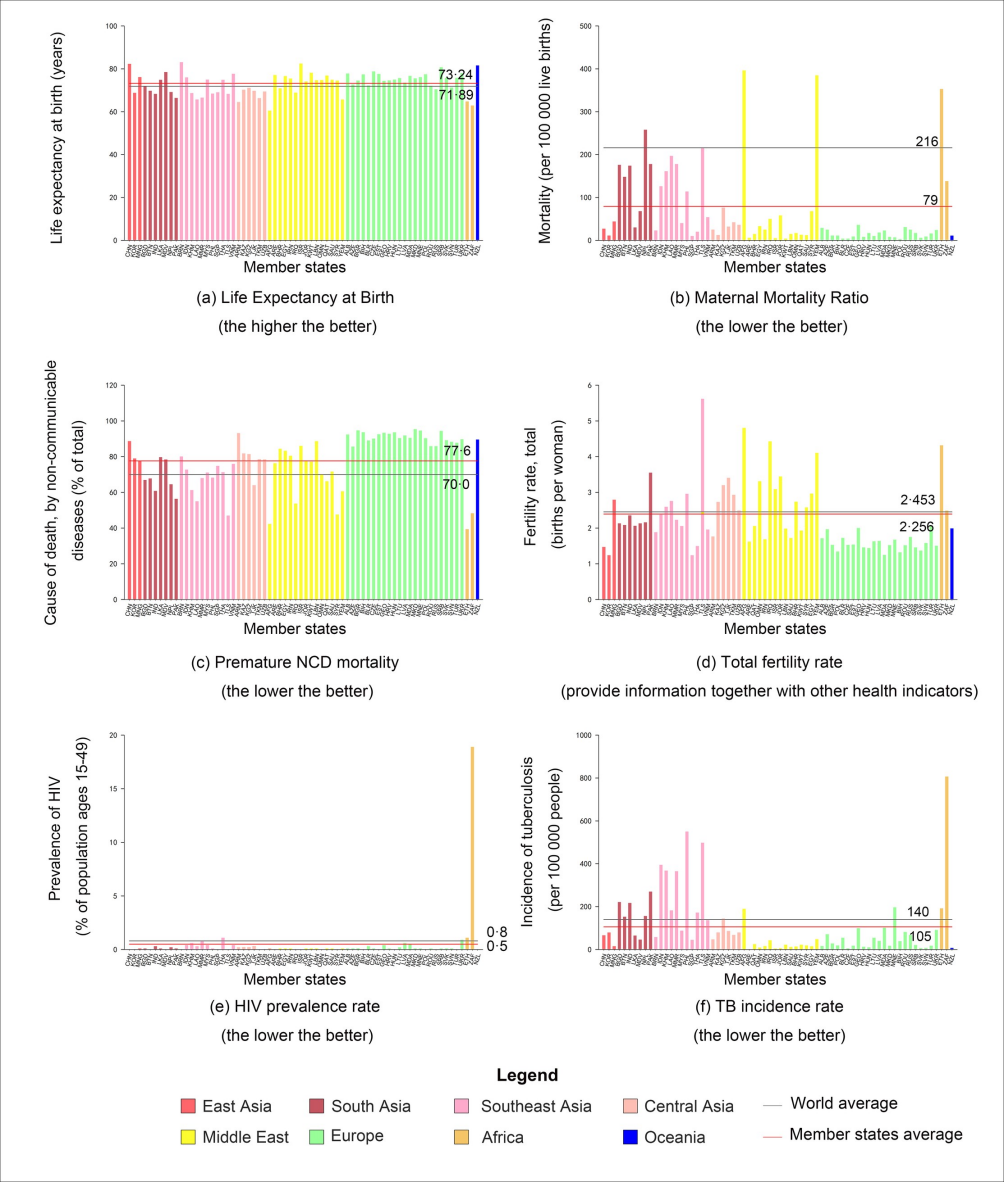
Health status as reflected by the selected health indicators in member states of the “belt and road” initiative in 2015.

In the first part, the exploratory spatial data analysis, we first examined the six selected health indicators for each member state and plotted histograms for each health indicator (Fig 2A–2F). The world average level of each health indicator was also plotted to facilitate comparison and used as a reference. Then we used global Moran's I to characterize spatial clustering or dispersion of the selected health indicators for all member states [17, 20]. Mathematically, Moran's I is expressed as:

$$I=\frac{n}{S_{0}}\frac{\sum_{i=1}^{n}\sum_{j=1}^{n}w_{i,j}z_{i}z_{j}}{\sum_{i=1}^{n}z_{i}^{2}}$$ (1)

Where $z_{i}$ is the deviation of an attribute for feature *i* from its mean $\;\left(x_{i}-\overset{-}{X}\right),\;w_{i,j}$ is the spatial weight between features *i* and *j*, *n* is the total number of features, and *S*_0_ is the aggregate of all the spatial weights.

$$S_{0}=\sum_{i=1}^{n}\sum_{j=1}^{n}w_{i,j}$$ (2)

The *Z*-score is:

$$Z\left(I\right)=\frac{1-\text{E}\left(I\right)}{\sqrt{V\left(I\right)}}$$ (3)

Where,

$$\text{E}\left(I\right)=\frac{-1}{n-1}$$ (4)

$$\text{V}\left(I\right)=E\left[I^{2}\right]-E{\left[I\right]}^{2}$$ (5)

Getis-Ord G* is employed to describe the spatial dependence and heterogeneity of the selected health indicators. Mathematically,

$$G_{i}^{\text{*}}=\frac{\sum_{j=1}^{n}W_{i,j}x_{j}-\bar{X}\sum_{j=1}^{n}w_{i,j}}{\sum_{j}^{n}W_{i,j}x_{j}}$$ (6)

Where *W*_i,j_ is the spatial weight between feature *i* and *j*. Positive $G_{i}^{\text{*}}$ value shows that high values cluster around *i* hence the region is termed “hot spot”, negative value of $G_{i}^{\text{*}}$ shows that low values cluster around *i* hence the region is termed “cold spot”.

In the second part of data analysis, we used a novel statistical method named “geographical detector” to examine if the stratified spatial heterogeneity found by visual inspection during exploratory spatial data analysis exist. Spatial stratified heterogeneity (SSH) refers to the phenomena that within strata are more similar than between strata [6]. Geographical detector is a tool to measure and find SSH of a variable Y, test the association between two variables Y and X, and investigate general interaction between two explanatory variables X1 and X2 to a dependent variable Y. SSH is measured in geographical detector by q-statistic, which measures the degree of spatial stratified heterogeneity and tests its significance. The q value is within [0,1] (0 if a spatial stratification of heterogeneity is not significant, and 1 if there is a perfect spatial stratification of heterogeneity) [6, 9]. There are four detectors included in geographical detector, namely [9]:

1. risk detector, which examines if there is a significant difference between the risks of different sub-regions by means of a t-test, measuring whether there is a significant difference between the means of two sub-regions having unequal variances.
2. factor detector, which measure whether a geographical stratum is responsible for an observed spatial pattern. It is calculated by the difference between the dispersion variance and the stratified population dispersion variance.
3. ecological detector, which determines whether a geographical stratum (associated with one suspected determinant) is more significant than another one (associated with another suspected determinant) in controlling the spread of the phenomenon concerned in space.
4. interaction detector, which measure whether two explaining factors, when taken together, weaken or enhance or independent with each another in developing the outcome of the dependent variable.

To examine the clustering of the selected health indicators visually apparent during exploratory data analysis, we used factor detector in geographical detector to examine if the continents a member states lies within influence the occurrence the values of the selected health indicators. In order to perform the analysis, we first divided each of the selected health indicators according to which continent it lies in, and then used factor detector to calculate the q-statistics for each selected risk factor. If the q-statistics was high, we regarded that the distribution of the health indicators resembled the distribution of the continents, and the continent a member states is in did play an important role in forming the health factors. If the q-statistics was low, we regarded that the distribution of the health indicators was not similar to the distribution of the continents, and the distribution of the continents did not influence the value of the health indicator. The value of the selected health indicators and the continents they lie in were fed into the geographic detector software package, which is based on Excel and can be downloaded free of charge at http://www.geodetector.org/.

### Data sources

The data used for the computation of the selected health indicators is based on the Global Health Observatory data (GHO) in year 2015 published by WHO. The data covered include global health priorities such as the health-related Millennium Development Goals, mortality and burden of disease, health systems, environmental health, noncommunicable diseases, infectious diseases, health equity and violence and injuries. In total, it contains health-related statistics for more than 1000 indicators for the 194 member states of WHO [21].

From the GHO data for all 194 member states of the WHO, we extracted data to only include the 68 member states of the “belt and road” initiative. Then, from more than 1000 indicators, we filtered out those that are necessary to calculate the six health indicators we have selected.

## Results

### Regional differences of the health indicators

Fig 2A–2F shows the selected health indicators (also shown in Table 2) for the member states, one for each indicator. Generally speaking, the health status of the member states, as indicated by the health indicators, dwindles around the world average level. Within the member states, the health status varies greatly. This is especially the case for noncommunicable diseases (represented by the premature non-communicable disease (NCD) mortality in Fig 2C) and infectious diseases (represented by the HIV prevalence rate in Fig 2E and the TB incidence rate in Fig 2F).

The geographic region of the “belt and road” initiative covers a vast area extending from Asia, Europe to Africa, the health status varies greatly for different geographic regions. European and Oceania member states generally have lower mortality and fertility rates, consequently higher life expectancy. On the other hand, member states in Africa and parts of Middle-East plagued by warfare (Afghanistan and Yemen) normally have higher mortality and fertility rates. Communicable disease prevalence such as HIV and TB are alarmingly high in South Africa when compared to other member states and the global average level and should be taken seriously.

The average life expectancy in the member states is slightly over one year higher than the world average (Fig 2A). Member states in Europe have a higher average life expectancy than others. In contrast, life expectancy in two member states in Africa are lower than the average of all member states and the world. Furthermore, variation of life expectancy among the member states is smaller compared with other indicators.

The average maternal mortality of member states is 137 people (per 100 000 births) less than the world average (Fig 2B). Member states in Europe and East Asia have lower mortality, while maternal mortality of member states in Africa and parts of Central Asia is higher. The variation of maternal mortality among member states is obvious. For example, Afghanistan has the highest maternal mortality (396 people per 100 000 births) while the lowest maternal mortality is only three people (per 100 000 births) in Poland.

The average premature NCD mortality of member states at 7.6% exceeds the world average (Fig 2C). It is the only indicator that shows a poorer health condition in member states when compared with the rest of the world. Specifically, premature NCD mortality of all states in Europe and Oceania exceeds the average of member states and the world average, while in Africa and part of the Central Asia, it is lower compared with other states.

The fertility rate in member states is approximately 0.2% lower than the world average, with obvious differences between member states (Fig 2D). European countries have the lowest fertility rates, and fertility rates in Africa and Central Asia are higher.

As for the HIV prevalence rate, even though the average of member states is only 0.3% lower than the world average, it is the result of averaging very high prevalence rate in African member states and very low prevalence rate in other member states (Fig 2E). For example, HIV prevalence in South Africa (18.9%) ranks the first, much higher than the rest of the member states. Even for Thailand and Ethiopia (1.1%) that ranks the second, the HIV prevalence in South Africa is more than 17 times.

The TB incidence rate in the member states is 35 people (per 100 000 population) lower than the world average, with obvious differences between member states (Fig 2F). In East Asia, Centre Asia, Middle East, Europe and Oceania, the TB incidence rate in most states are lower than the member states and the world average. However, for many states in Africa, Southeast Asia, and South Asia, the TB incidence rate is higher.

### Spatial distribution of the health indicators

Table 3 shows that the spatial distribution of all the selected health indicators has a positive spatial autocorrelation, which means that there is a high-value aggregation, i.e., the “hot spots” and low-value aggregation, i.e., the “cold spots”. The six indicators show different degrees of the spatial aggregation and dispersion (Table 3).

**Table 3:** Moran’s I of the health indicators of the member states.

**Health indicator**	**Moran’s I**	**Z-score**	**P-value**
Life expectancy at birth	0.143691	4.787981	0.000002
Maternal mortality ratio	0.160599	5.460206	0.000000
Premature NCD mortality	0.351363	11.075351	0.001096
Total fertility rate	0.199708	6.588791	0.000000
HIV prevalence rate	0.016568	2.193540	0.028268
TB incidence rate	0.215629	7.373296	0.000000

The P-values and Z-scores of the selected health indicators give information on their spatial distribution. The P-values of all health indicators are lower than 0.05, consequently we reject the null hypothesis that the distribution of the health level is generated by random processes and regard the distribution as dispersed or clustered. In addition, positive Z-sores implies that the distribution of all the health indicators are clustered rather than dispersed, that is, high values are distributed around the high values to form “hot spots” while low values cluster around low values to form “cold spots”. This statistical result coheres with the result from visual inspection that the health indicators are clustered, furthermore, it gives quantitative information as to the degree of clustering for each health indicator. The ubiquitous positive Z-scores and significant P-values show that within certain geographic region at continental scale, the health status as reflected by the health indicators exhibit a relative stable pattern, e.g., member states in Europe generally have better health status than those in Africa.

**Fig 3:**
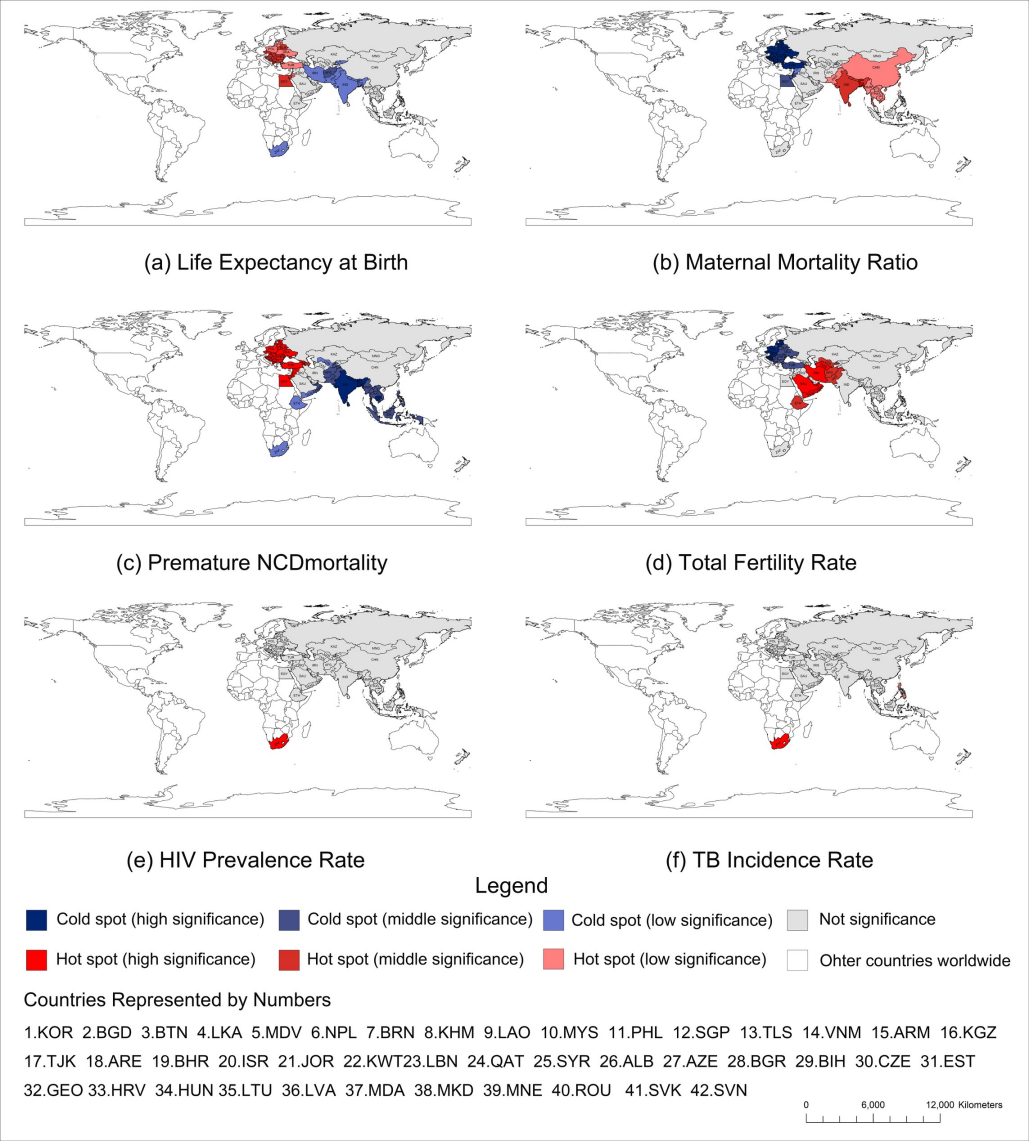
Getis-Ord G* of the health status of the belt and road initiative.

Getis-Ord Gi* can further detect these "cold spots" and "hot spots", and divide them into high-significant spots, medium-significant spots and low-significant spots. Fig 3 is a hot and cold spot detection map for the health status of the member states. Table 4 provides statistics about the number of cold spots and hot spots among the “belt and road”.

**Table 4:** Statistics of the cold spots and hot spots of health status among the member states.

Health status	Spots	Asia				Middle east	Europe	Africa	Oceania
		East Asia	South Asia	Southeast Asia	Central Asia				
Life expectancy at birth	Cold	6				3	0	1	0
		0	5	1	0				
	Hot	0				3	19	0	0
		0	0	0	0				
Maternal mortality ratio	Cold	1				5	21	0	0
		0	0	0	1				
	Hot	15				0	0	0	0
		2	8	5	0				
Premature NCD mortality	Cold	19				5	0	2	0
		0	7	10	2				
	Hot	1				5	22	0	0
		0	0	0	1				
Total fertility rate	Cold	0				0	20	0	0
		0	0	0	0				
	Hot	4				9	1	1	0
		0	0	0	4				
HIV prevalence rate	Cold	0				0	0	0	0
		0	0	0	0				
	Hot	1				0	0	1	0
		0	1	0	0				
TB incidence rate	Cold	0				0	0	0	0
		0	0	0	0				
	Hot	0				0	0	1	0
		0	0	1	0				

Cold spots are shaded blue. Hot spots are shaded red. And the color deepens as the amounts of the spots increasing.

For different indicators, cold spots and hot spots represent different health status. For life expectancy at birth, cold spots means shorter life span, and this generally imply lower health status. On the contrary, for maternal mortality, premature NCD mortality, HIV prevalence rate and TB incidence rate, cold spots generally means fewer incidence/prevalence and signifies higher health status. However, health status is a result of complex interaction between different factors, thus health indicator should be considered as reflecting different aspects of the health status and viewed together to get more insight of the data.

Fig 3A shows that there are no high-significant cold spots or hot spots for life expectancy. In the 32 medium-significant regions, "hot spots" include 22 countries in Europe, while “cold spots” are mainly distributed in the Middle East and South Asia, indicating that life expectancy in Europe is higher overall but in South Asia and Middle East more low-value life expectancy are clustered.

Fig 3B shows that for maternal mortality, most of the cold spots are located in Europe and Middle-East, signifying the low maternal mortality. Furthermore, cold spots in Europe are all high-significant, indicating a better maternal health condition in Europe. Hot spots of maternal mortality are mostly in Asia, indicating that maternal health status in Asia is relatively low.

From Fig 3C one observe that there are more hot and cold spots for premature NCD mortality compared with other health indicators, indicating a more clustered pattern. The hot spots are mostly concentrated in Europe, suggesting that more people died from non-communicable disease. However, this is not to say that the health conditions in Europe is inferior than other places. On the contrary, higher NCD mortality rate probably signifies better health status, especially when the life expectancy of the country is also high. If we have further information on maternal mortality ratio and incidence rate for infectious disease, we could make more accurate judgement as to the health status of a country. Take member states in Europe as an example, although the NCD mortality rate is high, but the life expectancy is also high (Fig 3A), at the same time, the maternal mortality ratio (Fig 3B) and the incidence of infectious diseases are low (Fig 3E and 3F). All the information derived from the health indicators enable us to conclude that member states in Europe have better health status than the rest.

Fig 3D shows that cold spots of fertility are all distributed in Europe, while the hot spots are mainly distributed in and around Middle East. The interpretation of total fertility rate should be combined with other health indicators, the absolute value of the total fertility rate alone can not show the health status of a given country.

Fig 3E shows there is only one hot spot of HIV prevalence, which is South Africa. Referring back to Fig 2, HIV prevalence rates in the two African member states ranked first and second, and much higher than the rest. The alarmingly high concentration of HIV incidence in member states in Africa shows that more international effort should take place to prevent HIV from spreading, especially for member states in “belt and road” initiative in the era of globalization. Fig 3F shows a similarly pattern of spatial distribution for TB incidence rates, except that there is also a medium-significant hot spot (PHL). Referring back to Fig 2, we find that member states in Africa and Southeast Asia suffer more from tuberculosis, especially South Africa and Philippine.

### Stratified spatial heterogeneity of the selected health indicators

The q-statistics and P-values of all the member states of the “belt and road” initiative are calculated by the geographical detector and the results shown in Table 5. The q-statistics are quantifications of the stratified spatial heterogeneity of the health indicators.

**Table 5:** Stratified spatial heterogeneity of all the member states of the “belt and road” initiative as quantified by q-statistics and P-values calculated by the geographical detector.

**Health indicator**	**q-statistic**	**p value**
Life expectancy at birth	0.269	1
Maternal mortality ratio	0.362	0.024
Premature NCD mortality	0.642	0.002
Total fertility rate	0.362	0.118
HIV prevalence rate	0.539	0.002
TB incidence rate	0.546	0.000

From Table 5, we can observe that life expectance at birth has relatively low q-statistic and the p-value is 1, which is far larger than the generally accepted threshold of 0.05, indicating that we can not conclude from this computation alone that life expectance at birth is clustered geographically. The same goes for total fertility rate with a q-statistic of 0.362 and p-value of 0.118. For the rest of the health indicators, the q-statistics are relatively higher, and the p-values are lower than 0.05, showing that they all cluster with respect to the sub(continents) the member states lie in. Generally speaking, mortality caused by non-communicable diseases (indicated by premature NCD mortality) and prevalence of communicable diseases (indicated by HIV prevalence rate and TB incidence rate) had large and significant q-statistics (q-statistics larger than 0.5 and p-value lower than 0.05), showed that the adverse effect of diseases, whether communicable or non-communicable, exhibited high level of clustering.

## Discussion

In order to measure the health status of certain countries in an objective, comprehensive yet concise, and universally applicable manner, the 2018 Global Reference List of 100 Core Health Indicators published by WHO were utilized. From which six health indicators were selected. Although the selection of the indicators has been put into careful consideration, it can be argued that the selection is subject to personal preference. This is especially the case for the selection of indicators for infectious disease. In this paper, we selected TB and HIV prevalence rate as indicators for morbidity of infectious diseases, although prevalence of other infectious diseases such as Hepatitis B, congenital syphilis, or malaria could also be used. The reason for choosing TB and HIV over other infectious diseases is their relatively higher rate of prevalence, worldwide occurrence (e.g., unlike malaria, which has higher prevalence rate in tropical regions, the distribution of TB occurrence is less dependent on geographic location), and higher reception for general public (e.g., HIV is probably more familiar for general public than congenital syphilis).

Despite the fact that only health factors in the “health status” category of the Global Reference List are selected as indications of the health status of the member states in the “belt and road” initiative, we argue that these indicators do imply other health indicators in the rest of three categories in the Global Reference List, as many of the indicators are interrelated to some extent. For example, “life expectancy at birth”, “maternal mortality ratio” and “premature noncommunicable disease mortality” we chose may reflect the nutrition, health service coverage and quality and safety of health care system attributed to other categories of health indicators. “maternal mortality ratio” could reflect the “utilization and access of health care services”, “health financing”, and “existence of national health sector policy/strategy/plan”. The same can also be stated for the prevalence rate of TB and HIV as indicators for the development of health systems and health care service.

It should be pointed out that although health status was quantified using various health indicators, direct comparison is not always sensible. For example, a life expectancy at birth of 1.35 year more for the member states than the world average (calculated by subtracting 73.24 years to 71.89 years) should not be interpreted as: on average, the people living in the member states are expected to live 1.35 years longer than people in the rest of the world. Many different factors are at work interactively, and their interaction is so complex that a statistic alone can not capture the whole process. Hence, multiple health indicators together provide us with more concrete and reliable information. The low life expectancy at birth (Fig 2A) together with very high HIV prevalence rate (Fig 2E) and TB incidence rate (Fig 2F) for African member states signify that a large proportion of the mortality are caused by the prevailing infectious diseases. Besides HIV and TB, it is suspected that other infectious diseases such as congenital syphilis rate and malaria incidence would also be high. Furthermore, despite that fact that the premature NCD rates for member states in Africa are relatively low (Fig 2C), this does not mean that the health status in member states in Africa are better than those in Europe, for example. On the country, it shows that the prevalence for infectious diseases in member states in Africa is so high, that people often died early as a result, such that death caused by NCDs (usually when people get much older) is relatively low. Moreover, from the relatively high maternal mortality ratio for the member states in Africa (Fig 2B), we can conclude that this also contributes to the low premature NCD mortality (as people have died at birth before they get a cardiovascular disease).

The values of q-statistics calculated using geographical detectors confirmed statistically the clues revealed by exploratory data analysis that the selected health indicators are clustered. The results given by exploratory data analysis using Moran’s I and Getis-ord Gi* and statistical model such as the geographical detectors complement each other. The results obtained by exploratory data analysis can be presented in the form of maps and can give intuitive sense of the distribution of the selected health indicators, while the results computed using statistical model such as the geographical detector give quantitative and statistically sound values for the degree of clustering.

The results of the q-statistics conform with our knowledge on the health status of the member states and the discussions we just presented. This is, for relatively simple health indicators such as the mortality rate and prevalence rate of diseases, whether communicable diseases or non-communicable diseases, the spatial pattern (clustering, dispersion or randomly distributed) generally shows a clear trend, while for complex health indicators such as life expectance at birth, maternal mortality ratio and total fertility rate, the spatial pattern and spatial stratified heterogeneity indicated by the q-statistics are not clearly put, as the value of the health indicators depend on other complicated statistics such as population, GDP, health facilities, and insurance systems.

Based on the research of six health indicators selected in this paper, the results suggested that the health status of the member states dwindled around the world average while the difference within the member states are high. In East Asia health status of member states are generally good. However the premature NCD mortality of all the three member states in East Asia are higher than the world and member state averages. Health status in South Asia is relatively lower. Life expectancy, maternal mortality, premature NCD mortality and TB incidence vary between member states. Some cold spots of life expectancy and hot spots of maternal mortality and HIV morbidity are located in South Asia. In Southeast Asia, the health status are generally good. However Myanmar, Cambodia, Vietnam, etc. that are close to South Asia have relatively lower levels of health status, with lower life expectancy and higher mortality and morbidity. The health level of member states in the Middle East varies. The health status there is generally high, with some exceptionally high maternal mortality ratios. Member states in Europe generally have good health status and low fertility rates. Similar pattern applies to the member state New Zealand in Oceania. There were two African member states, both of them have inferior health status, especially for infectious diseases such as HIV and TB.

Examining the regional differences and spatial patterns of the health status of member states can help us to measure the overall health status of member states and provide an objective and common reference for the comparison of the health status globally. The findings for the alarmingly high incidence rate for member states in Africa calls for urgent countermeasure to improve the health conditions and prevent the infectious diseases from spreading. This is especially important for member states in the “belt and road” initiative. Further research is needed for underlying driver of the spatial patterns and possible measures to improve the health status in African member states.
